# A Hierarchical Network Approach for Modeling Rift Valley Fever Epidemics with Applications in North America

**DOI:** 10.1371/journal.pone.0062049

**Published:** 2013-05-07

**Authors:** Ling Xue, Lee W. Cohnstaedt, H. Morgan Scott, Caterina Scoglio

**Affiliations:** 1 Kansas State Epicenter, Department of Electrical and Computer Engineering, Kansas State University, Manhattan, Kansas, United States of America; 2 Center for Grain and Animal Health Research, United States Department of Agriculture, Manhattan, Kansas, United States of America; 3 Department of Diagnostic Medicine/Pathobiology, Kansas State University, Manhattan, Kansas, United States of America; Louisiana State University, United States of America

## Abstract

Rift Valley fever is a vector-borne zoonotic disease which causes high morbidity and mortality in livestock. In the event Rift Valley fever virus is introduced to the United States or other non-endemic areas, understanding the potential patterns of spread and the areas at risk based on disease vectors and hosts will be vital for developing mitigation strategies. Presented here is a general network-based mathematical model of Rift Valley fever. Given a lack of empirical data on disease vector species and their vector competence, this discrete time epidemic model uses stochastic parameters following several PERT distributions to model the dynamic interactions between hosts and likely North American mosquito vectors in dispersed geographic areas. Spatial effects and climate factors are also addressed in the model. The model is applied to a large directed asymmetric network of 3,621 nodes based on actual farms to examine a hypothetical introduction to some counties of Texas, an important ranching area in the United States of America. The nodes of the networks represent livestock farms, livestock markets, and feedlots, and the links represent cattle movements and mosquito diffusion between different nodes. Cattle and mosquito (*Aedes* and *Culex*) populations are treated with different contact networks to assess virus propagation. Rift Valley fever virus spread is assessed under various initial infection conditions (infected mosquito eggs, adults or cattle). A surprising trend is fewer initial infectious organisms result in a longer delay before a larger and more prolonged outbreak. The delay is likely caused by a lack of herd immunity while the infection expands geographically before becoming an epidemic involving many dispersed farms and animals almost simultaneously. Cattle movement between farms is a large driver of virus expansion, thus quarantines can be efficient mitigation strategy to prevent further geographic spread.

## Introduction

Rift Valley fever (RVF) was first identified in Egypt in 1931 [1] and is endemic in the eastern and southern regions of Africa [2]. Viral infection may result in abortion in adults and death in newborn livestock [3]. Sheep, goats and cattle are the most important domestic animal hosts affected when viewed from an economic standpoint [2] although humans also can become infected [3], [4].

*Aedes* and *Culex* genera of mosquitoes are thought to be main RVF disease vectors with respect to vector competence [4]. The virus is maintained between epidemics through vertical transmission within the *Aedes* mosquitoes [5], and is thought to be propagated and amplified during epidemics by both *Aedes* and *Culex* species mosquitoes. High RVF transmission is typically related to persistent, above average rainfall and El Niño/Southern Oscillation (ENSO) events in Eastern Africa which create favorable mosquito habitats [6]. *Aedes* mosquitoes lay eggs in dry mud [7] and the eggs can survive for long periods of time [2]. After flooding, RVF virus-infected eggs can develop into infected adult mosquitoes [2]. Infected adult *Aedes* mosquitoes then feed on animals which become infected, and spread the infection to other *Aedes* and *Culex* genera adult mosquitoes feeding on infected animals.

Animal movements, typically motivated by livestock trading and marketing may accelerate the transmission of zoonotic diseases among animal holdings which may cover a vast area [8]. In 1977, the trade of sheep from east Africa during Ramadan was considered to be a likely pathway for the introduction of RVF virus to Egypt [9]–[11]. A boy from Anjouan, an island of Comoros archipelago, was diagnosed to have been infected with RVF virus on the French island of Mayotte in 2007 [4]. The Rift Valley fever virus was likely to be introduced by live ruminants imported from Kenya or Tanzania in the trade during the 2006–2007 Rift Valley fever outbreak [4].

Humans can acquire the infection from the bites of infected mosquitoes or directly from contact with the bodily fluids of infected animals [12]. Individuals working with animals, such as farmers and veterinarians, are the most vulnerable to RVF virus infection during animal outbreaks [13] because of increased exposure to mosquitoes in an outdoor environment and direct contact with animals. Rift Valley fever virus infection causes severe influenza-like disease in humans with serious consequences such as blindness, or even death [3]. It has been reported that more than 200 persons died of RVF in Mauritania in 1987 [14]. There were 738 reported human cases in Sudan, including 230 deaths, in 2007–2008 [15]. It is likely that the number of human cases has been underreported in the past, especially in rural areas [4]. Rift Valley fever virus has spread outside of Africa to Yemen and Saudi Arabia in 2000 [4] and the French island of Mayotte with multiple human cases reported [16]. Rift Valley fever virus could possibly be introduced to the United States, similar to the experience with West Nile virus which was introduced into the North America in 1999 [17]. A mathematical epidemiological model can be applied to non-traditional locations in order to study the potential for spatial spread of RVF virus.

Epidemiological modeling plays an important role in planning, implementing, and evaluating detection, control, and prevention programs [18]. Mathematical modeling takes the advantage of economic, clear and precise mathematical formulation, e.g., applications of differential, integral, or functional differential equations [18]. Mathematical models of infection transmission include interpretation of transmission processes and are often useful in answering questions that cannot be answered only with empirical data analysis [19], as well as to explore biological and critical ecological characteristics of disease transmission [20], [21]. Current RVF virus transmission models are useful in representing infection transmission process [19] but are limited in determining and testing relevant risk factors. For the Ferlo area of Senegal, a pond-level meta-population model which considered only vectors was developed assuming that *Aedes* mosquitoes were the only vector and rainfall was the only driving force [2]. It has been shown that within Ferlo, the virus would persist only if the livestock moved between ponds and the rainfall did not occur in all ponds simultaneously [2]. Very few mathematical dynamic transmission models have explored mechanisms of RVF virus circulation [19] on a larger geographical scale. A theoretical model in a closed system including *Aedes* and *Culex* mosquitoes and livestock population was earlier proposed [22]. The key result was that RVF virus can persist in a closed system for 10 years if the contact rate between hosts and vectors is high [19], [22]. Another theoretical RVF virus transmission mathematical model [23] modified the model in [22] by adding human hosts, merging all mosquitoes into one class, removing mosquito egg compartment, as well as vertical transmission of mosquitoes. Sensitivity indices of the reproduction number are used to determine the most sensitive parameters to the basic reproduction number of RVF virus transmission [23]. It has been found that both the reproduction number and disease prevalence in mosquitoes are sensitive to mosquito death rate and the disease prevalence in livestock and humans are more sensitive to livestock and human recruitment rates [23]. A theoretical ordinary differential equation meta-population involving livestock and human mobility was presented [24]. They analyzed the likelihood of pathogen establishment and provided hypothesized examples to illustrate the methodology [24]. A three-patch model for the process by which animals enter Egypt from Sudan, are moved up the Nile, and then consumed at population centers is proposed [25]. Using [22] and [23] as a foundation, the homogeneous models have been extended to a meta-population differential equation model including *Aedes*, *Culex*, livestock, and humans and a case study was carried out for South Africa during a country-wide outbreak in 2010 [26]. The model was based on RVF virus spatial transmission during an outbreak, where a network with three nodes corresponding to three affected provinces in South Africa was established. To make the output of the model [26] easily compared with incidence data if available and the simulation for thousands of nodes easily implemented, a discrete time epidemic model is developed and a much larger network on which to study the dynamics of the larger system is established.

Proposed here is a deterministic network-based RVF virus transmission model with stochastic parameters. Two competent vector populations: *Aedes* mosquitoes, *Culex* mosquitoes, and two host populations: cattle and humans are considered. The dynamical behavior of mosquito and livestock populations are modeled using a meta-population approach based on weighted contact networks. The nodes of the networks represent geographical locations, and the weights represent the level of contact between regional pairings. In particular, nodes represent different farm sizes or operator businesses of the cattle industry, nominally markets and feedlots. Heterogeneous aspects of the spreading are considered in the model through realistic modeling of the cattle movement among different nodes of the network. Additionally, the mosquito population and development is modeled as a function of climatic factors, such as humidity and temperature. It is easy to implement simulations of the model even for networks with thousands of nodes, and it is easy to compare the output of the model with incidence data if available. The role of starting location has been shown to be important in the final size of rinderpest epidemic [27]. To investigate the role of starting location, and the size of initial infection in RVF virus spread, the proposed model has been applied to a case study to some counties in Texas, U.S. and the model outcomes (the human and cattle cases, and the timing of the epidemic’s characteristics) indicate which biotic factors will play an important role if RVF virus is introduced to the United States.

## Materials and Methods

### Network-based Meta-population Models

**Figure 1:**
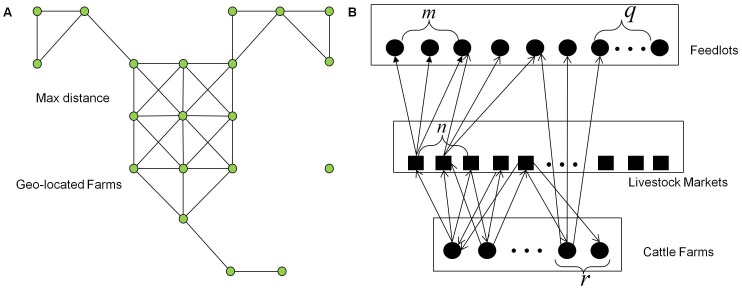
Network illustration. (A) A hypothetical mosquito diffusion network demonstrating how mosquito move to farms that are smaller than 2 km away. (B) Livestock move bidirectionally between livestock farms and livestock markets but only move unidirectionally to feedlots as demonstrated in the livestock movement network.

*Aedes* mosquitoes, *Culex* mosquitoes, livestock, and human populations each are considered in the network-based meta-population models. The movement of each population is represented by networks, where nodes denote locations, and links denote movement flow between locations. In the mosquito diffusion network, the nodes represent farms and the links represent mosquito diffusion from one farm to the neighboring farms. The weights are diffusion rates 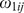 for *Aedes* population, and 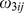 for *Culex* population from location 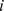 to location 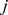. In the livestock movement network, the nodes represent farms, livestock markets, and feedlots. The links represent livestock movements due to livestock trade between the nodes and the weight is the movement rate 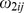 from node 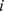 to node 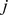. The mosquito and livestock networks are shown in Fig. 1A and Fig. 1B, respectively.

The compartmental models are adapted to represent the status of each population during a simulated RVF virus transmission. The models are built based on the principle of the RVF virus transmission flow diagram illustrated in [26]. Adult *Aedes* and *Culex* populations are distributed among susceptible 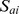, exposed 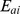, and infected 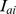 compartments. Only those mosquito species that are known to be competent vectors of RVF virus transmission are considered and they are broadly grouped by *Aedes* and *Culex* genera mosquitoes. The subscript 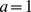 denotes *Aedes* in node 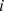, and 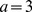 denotes *Culex* mosquitoes in node 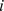. Uninfected and infected mosquitoes eggs are represented by 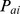 and 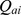, respectively. *Culex* mosquitoes do not display vertical transmission. Therefore, only uninfected *Culex* eggs are incorporated in the model. The livestock and human hosts are likewise considered 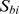, 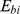, 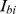, and 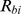. The subscript representing livestock in node 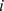 is 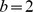, and humans in node 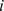 are represented with 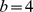. The descriptions of the parameters in the models are found in Table 1. All the transitions to be discussed below are for location 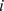 at day 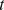.

**Table 1:** Parameter ranges for numerical simulations.

Para-meter	Description	Range	Assumed most possible value	Units	Source
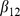	contact rate: *Aedes* to livestock	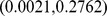	0.1392	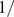 day	[43]–[49]
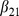	contact rate: livestock to *Aedes*	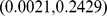	0.1225	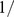 day	[43]–[47], [50]
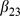	contact rate: livestock to *Culex*	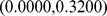	0.16	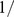 day	[44]–[47], [50], [51]
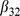	contact rate: *Culex* to livestock	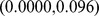	0.04	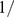 day	[44]–[47], [51]
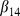	contact rate: *Aedes* to humans	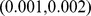	0.0015	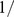 day	Assume
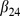	contact rate: livestock to humans		0.00006	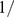 day	Assume
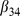	contact rate: *Culex* to humans	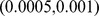	0.000525	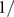 day	Assume
	recovery period in livestock	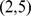	3.5	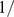 day	[52]
	recovery period in humans	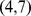	5.5	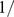 day	[23]
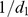	longevity of *Aedes* mosquitoes		31.5	days	[47], [53], [54]
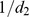	longevity of livestock	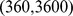	1980	days	[55]
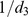	longevity of *Culex* mosquitoes		31.5	days	[47], [53], [54]
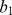	birth rate of *Aedes* mosquitoes	weather dependent		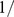 day	[35]
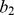	birth rate of livestock	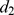		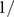 day	[55]
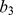	birth rate of *Culex* mosquitoes	weather dependent		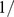 day	[35]
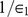	incubation period in *Aedes* mosquitoes	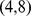	6	days	[48]
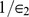	incubation period in livestock	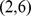	4	days	[56]
	incubation period in *Culex* mosquitoes	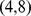	6	days	[48]
	incubation period in humans	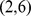	4	days	[23]
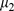	mortality rate in livestock	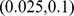	0.0375	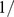 day	[52], [56]
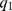	transovarial transmission rate in *Aedes* mosquitoes	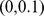	0.05	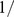 day	[57]
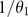	development period of *Aedes* mosquitoes	weather dependent		days	[36]
	development period of *Culex* mosquitoes	weather dependent		days	[35]
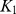	carrying capacity of *Aedes* mosquitoes				Assume
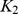	carrying capacity of livestock		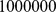		Assume
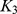	carrying capacity of *Culex* mosquitoes		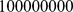		Assume
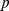	reduction in 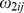 due to infection		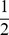		Assume

### *Aedes* Population Model



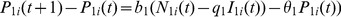
(1)

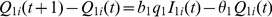
(2)

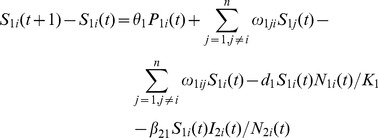
(3)

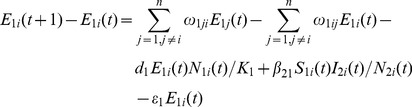
(4)

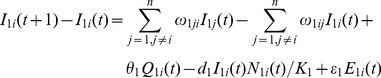
(5)

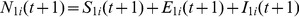
(6)


There are 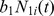 eggs laid, including 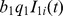 infected eggs, and 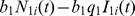 uninfected eggs each day. After the development period, 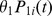 uninfected eggs develop into susceptible adult *Aedes* mosquitoes and 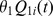 infected eggs develop into infected adult *Aedes* mosquitoes. The number of *Aedes* mosquitoes infected by livestock is 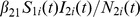. Following the incubation period, 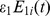 *Aedes* mosquitoes transfer from exposed compartment to infected compartment. The number of *Aedes* mosquitoes dying naturally in compartment 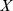 is given as 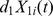. The percentage of *Aedes* mosquitoes moving from location 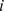 to location 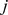 is 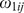. The change in the number of *Aedes* mosquitoes due to mobility in compartment 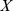 is given as 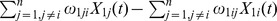 [28].

### *Culex* Population Model



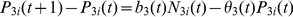
(7)

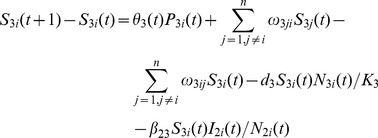
(8)

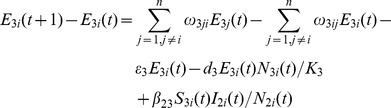
(9)

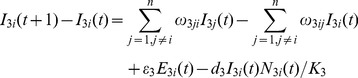
(10)

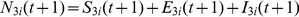
(11)


There are 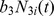 eggs laid each day. After the development period, 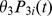 eggs develop into susceptible adult *Culex* mosquitoes. After the incubation period, 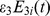 *Culex* mosquitoes transfer to infected compartment 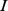. The number of *Culex* mosquitoes acquiring infection from livestock is denoted by 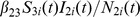. The *Culex* mosquitoes removed from compartment 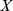 due to natural death is 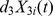. The percentage of *Culex* mosquitoes moving from location 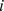 to location 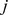 is 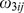. The change in the number of *Culex* mosquitoes due to movement in compartment 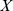 is given as 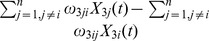 [28].

### Livestock Population Model



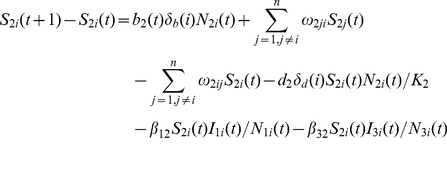
(12)

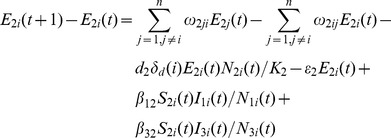
(13)

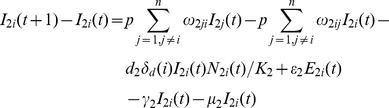
(14)

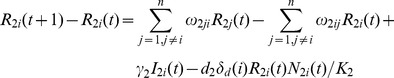
(15)

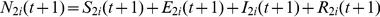
(16)


The daily number of newborn livestock in location 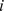 is 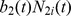. The variables 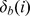 and 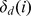 are used to differentiate different types of nodes. If location 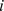 is a farm, then 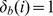, 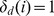. If location 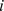 is a market, then 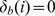, 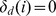. If location 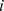 is a feedlot, then 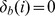, 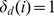. The numbers of livestock infected by *Aedes* mosquitoes and *Culex* mosquitoes are denoted by 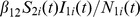 and 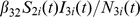, respectively. After the incubation period, 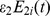 livestock transfer from exposed state to infected state. After the infection period, 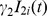 livestock recover from RVF virus infection. The number of dead livestock in compartment 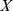 is given as 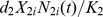 in which 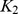 is the carrying capacity of livestock in each node. The change in the number of livestock in compartment 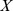 due to mobility is given as 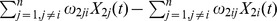 for livestock in compartments 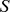, 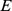, and 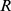, and 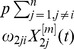-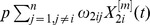 [28], 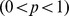 for livestock in compartment 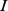.

### Human Population Model



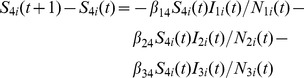
(17)

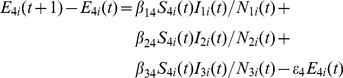
(18)

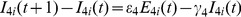
(19)

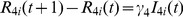
(20)


The number of humans in each node is constant because birth, death, mortality, and mobility of humans are not considered. The number of humans infected by *Aedes* mosquitoes, *Culex* mosquitoes, and livestock is 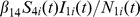, 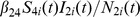, and 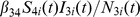, respectively. There are 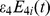 humans transferring to infected compartment after incubation period, and 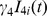 humans recovering from RVF virus infection after infection period.

### Case Study: Texas, U.S.A. from 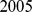 to 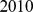


#### Networks in the study area

As a case study, various RVF virus introduction scenarios were tested using the model to determine the hypothetical model outcomes (number of livestock cases and timing of the epidemic). Although the model accounts for their exact locations when simulating RVF virus spread, we do not report any of this information or even discuss ranches in areas smaller than county level. The exact farms and counties are very well masked from the results. Texas cattle ranches were selected because they have large cattle concentrations and we have aggregate survey data on cattle movements in these areas [29]. A network with 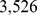 cattle farms [30], 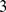 livestock markets [30], and 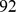 cattle feedlots [30] is constructed. The cattle farms, and livestock markets are located in one region, and the feedlots are in another region. The location of each node is uniformly distributed in each county according to the total number of farms within each county [30]. The exact location of each farm is obscured because those data are not publicly available [31] due to confidentiality. The initial number of cattle in each farm is categorized as 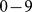, 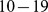, 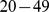, 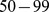, 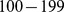, 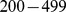 and more than 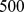 [30]. The initial number of susceptible cattle in each farm or feedlot for numerical simulation is assumed according to the number of cattle in each county in 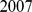 [30] and the histogram of the number of cattle [30]. For cattle movement, if cattle are sold from one node to another, then there is a link between the nodes. The movement rate of cattle denoted by 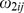 shown in Table 2 is estimated based on the aggregate movement rates from survey [29] and inversely proportional to the distance between source-destination pairs. Movement rate is the average movement rate for all cattle at different ages, and the movement rate of cattle in compartment 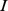 is assumed to be half the movement rate for cattle in compartments S, E, and R, namely, 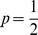.

**Table 2:** Cattle movement rate 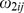, where 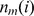 = the number of markets connected to farm 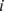, 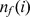 = the number of farms connected to market 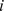, 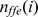 = the number of feedlots connected to farm 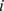, 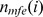 = the number of feedlots connected to market 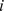.

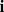	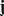	Range	Source
farm	market	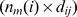 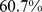 /	[29]
market	farm	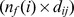 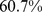 /	[29]
farm	feedlot	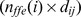 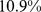 /	[29]
market	feedlot	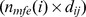 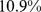 /	[29]
feedlot	farm	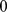	[29]
feedlot	market	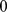	[29]

For mosquito diffusion, if the distance between two farms is smaller than an assumed radius, two kilometers, then there is a link between the nodes in the network. The diffusion rates of *Aedes* and *Culex* mosquitoes are shown below [32].
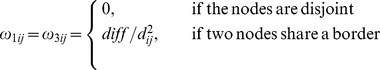
where 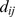 is the distance between the centers of node 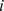 and node 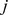 [32] and 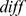 is a diffusion like parameter within the range 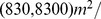
day [32].

#### Parameters for numerical simulations

Vector competence varies within and between mosquito species [33]. Stochastic parameters were used to account for broad range of vector competence between *Aedes* and *Culex* species and individual variation within each species. The PERT distribution has few constraints (minimum, maximum, and most likely value), similarly to the triangular distribution applied in [34] to simulate West Nile virus epidemic. In the following simulations, PERT distributions are selected to generate stochastic parameters with ranges and the most likely values listed in Table 1. Any appropriate parameter distribution can be adapted into the model.

**Figure 2:**
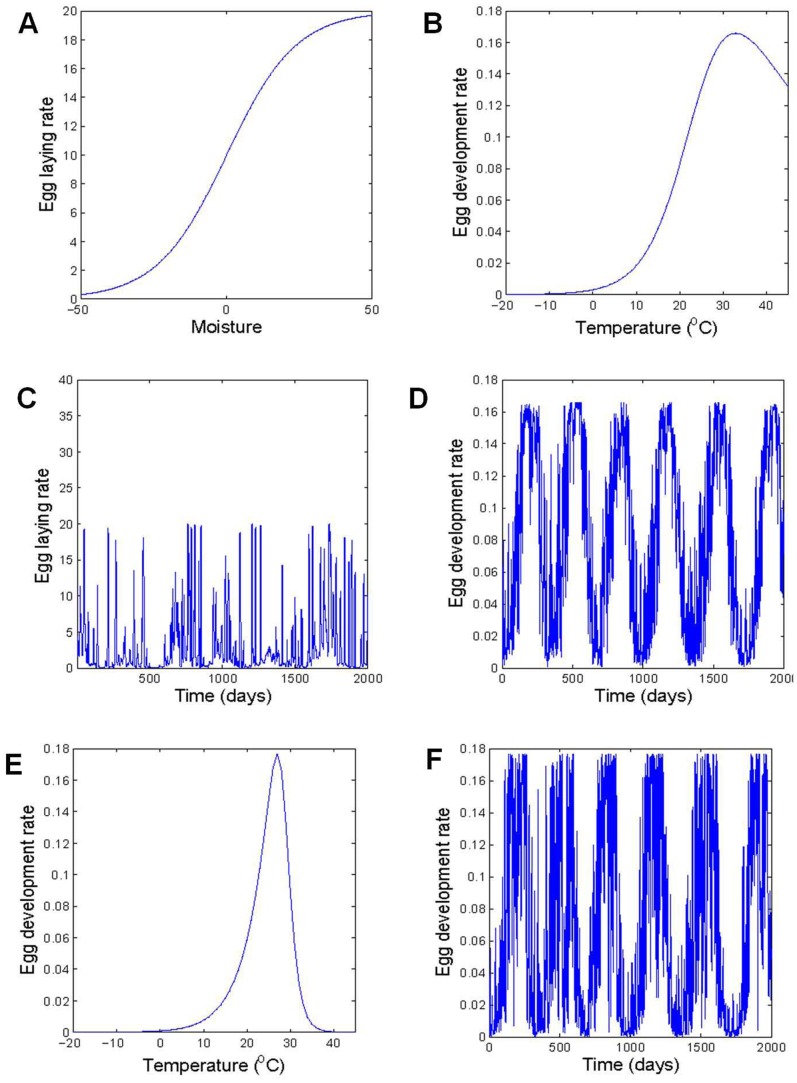
The relationship between egg laying rates, egg development rates of mosquitoes and climate factors. (A) The egg laying rates of *Aedes* and *Culex* mosquitoes with moisture [35]. (B) The egg development rate of *Culex* mosquitoes with temperature [35]. (C) The egg laying rates of *Aedes* and *Culex* mosquitoes in the nine counties in the south of Texas from January, 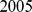 to October, 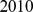. (D) The egg development rate of *Culex* mosquitoes in one region of Texas from January, 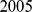 to October, 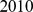. (E) The egg development rate of *Aedes* mosquitoes with temperature. (F) The egg development rate of *Aedes* mosquitoes in one region of Texas from January, 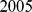 to October, 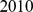.

The egg laying rates of *Aedes* and *Culex* mosquitoes changing with moisture conditions as indicated in Equation (21) [35] are shown in Fig. 2A. The egg development rate of *Aedes* mosquitoes varying with temperature in Equation 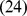 [36] and that of *Culex* mosquitoes in Equation 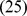 are in Fig. 2E and Fig. 2B, respectively. The parameters for egg laying rates of *Aedes* mosquitoes and *Culex* mosquitoes, and parameters for egg development rate of *Culex* mosquitoes are derived from data concerning West Nile virus in 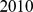 in the Northern U.S. [35], and the parameters for the egg development rate of *Aedes* mosquitoes is derived using the model for *Aedes* aegypti [36], which are the best models currently available. More precise parameters can be adopted, as they become available. The egg laying rates of *Aedes* and *Culex* mosquitoes, egg development rate of *Culex* mosquitoes, and egg development rate of *Aedes* mosquitoes computed with the climate data for the region where cattle farm and markets located in the study area of Texas from January, 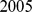 to October, 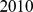, are shown in Fig. 2C, Fig. 2D, and Fig. 2F, respectively. If the temperature is too low, the eggs will not develop into larvae and then adult mosquitoes. If the temperature is too high, the lifespan of the mosquitoes is shortened and the development rate decreases. Moisture index is the difference between precipitation and evaporation as shown in Equation (22). A lower moisture index correlates to fewer adult mosquitoes because low moisture index represents a combination of low precipitation and high evaporation. For some days, the missing precipitation data from January, 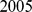 to December, 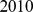 [37] are assumed to be zero. The evaporation data are calculated using Equation (23) [38]. The parameters in Equations (21) through (25) are listed in Table 3. Although humans move between nodes, they do not transmit virus between nodes and the number of humans in each node (i.e., farm) is assumed to be fewer than 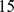.

**Table 3:** Parameters in Equations (21) through (25).

Parameter	Description	Value	Source
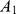	parameter in Equation (24)	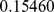	[36]
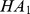	parameter in Equation (24)	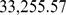	[36]
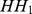	parameter in Equation (24)	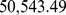	[36]
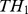	parameter in Equation (24)	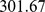	[36]
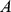	parameter in Equation (25)	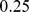	[35]
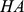	parameter in Equation (25)	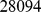	[35]
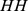	parameter in Equation (25)	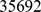	[35]
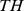	parameter in Equation (25)	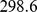	[35]
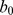	minimum constant fecundity rate	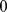	[35]
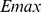	maximum daily egg laying rate	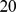	[35]
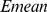	the mean of the daily egg laying rate	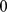	[35]
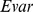	variance of function	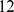	[35]



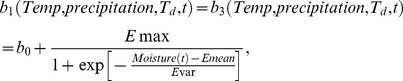
(21)

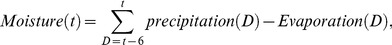
(22)

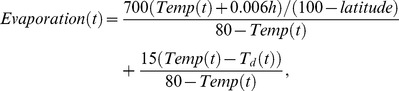
(23)

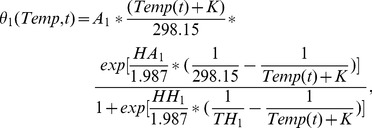
(24)




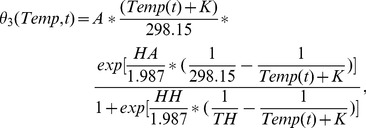
(25) where


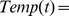
air temperature in units of 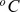 [38].


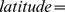
the latitude (degrees) [38].


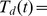
the mean dew-point in units of 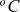 [38].


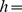
the elevation (meters) [38].


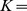
Kelvin parameter.

## Results

### The Novel Mathematical Model

Presented is a discrete time compartmental mathematical model based on a network approach. Rift Valley fever is transmitted by several species of mosquito vectors that have varying levels of vector competence; therefore, each genus and species combination requires modeling the vector competence, movement, and life stage development patterns which is too complicated while considering only a single species or genus is not accurate. Consequently, the species are loosely grouped as their genera and the parameters are allowed to vary following PERT distributions. The distribution captures uncertainties on inherent variability between species, as well as variability among individual mosquitoes. The mosquito parameters are functions of climate factors to reflect the impact of climate and season on mosquito dynamics. Only *Aedes* and *Culex* genera mosquitoes that are competent vectors of RVF virus transmission are considered in the model.

Different networks are developed for mosquito diffusion and livestock movement considering heterogeneity in both. In the cattle movement network, different types of nodes distinguish between sources, sinks, and transitions.

The model can be used to simulate networks with the number of nodes up to thousands with the easily solvable discrete time model. To use the model in any location, one only needs the initial populations, the movement rates, ranges of the parameters, and climate factors in each location to obtain the epidemic curve.

### Case Study

Sixteen initial conditions shown in Table 4 in two regions of Texas, U.S.A. from January 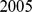 to October, 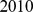 were tested with the model to determine their effects on the simulated and hypothetical spread of Rift Valley fever virus were it to be introduced. The average results of 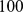 realizations for each scenario starting in the same small or large farm are presented qualitatively in Table 5, and the quantitative numerical simulation results are shown in the Supporting Information section. For the simulations an introduction to a small farm is a farm with fewer than 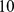 cattle and the large farm is considered a farm with more than 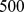 cattle. By changing initial locations in extensive numerical simulations, we obtained different value for each variable from that of corresponding one in the table of Supporting Information but similar trends. Note at this time no specific mitigation strategies are applied here; during an outbreak the RVF virus control methods post detection will be expected to modify any such results.

**Table 4:** Sixteen different initial conditions.

Farm size	Quantity			Infected	
		*Aedes* eggs	*Aedes* mosquitoes	*Culex* mosquitoes	Cattle
Small	Few	*Aedes* -eggs-f-s	*Aedes* -f-s	*Culex* -f-s	Cattle-f-s
	Many	*Aedes* -eggs-m-s	*Aedes* -m-s	*Culex* -m-s	Cattle-m-s
Large	Few	*Aedes* -eggs-f-l	*Aedes* -f-l	*Culex* -f-l	Cattle-f-l
	Many	*Aedes* -eggs-m-l	*Aedes* -m-l	*Culex* -m-l	Cattle-m-l

**Table 5:** Qualitative numerical simulation results of different scenarios with respect to infected cattle.

			Initial	source	of	infection
Farm size	Initial infection size	Outcome characteristics	*Aedes* eggs	*Aedes* adult	*Culex* adult	Cattle
Small	Few ( 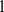 )	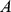	average	small	very small	very small
		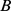	very large	very large	large	average
		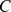	very large	very large	average	very small
		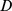	very long	very long	long	medium
		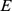	medium	long	very long	short
	Many ( 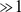 )	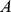	very small	large	very large	average
		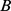	average	small	very small	small
		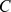	very small	small	average	very small
		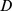	short	short	short	short
		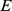	short	very short	very short	very short
Large	Few ( 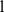 )	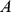	very small	very small	very small	small
		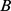	very large	large	average	very large
		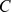	very small	small	very small	average
		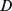	long	long	short	very long
		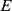	very long	medium	short	long
	Many ( 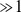 )	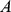	very large	very large	very large	very small
		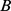	very small	small	small	large
		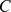	average	large	average	small
		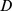	short	very short	very short	long
		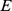	very short	short	short	medium

**Figure 3:**
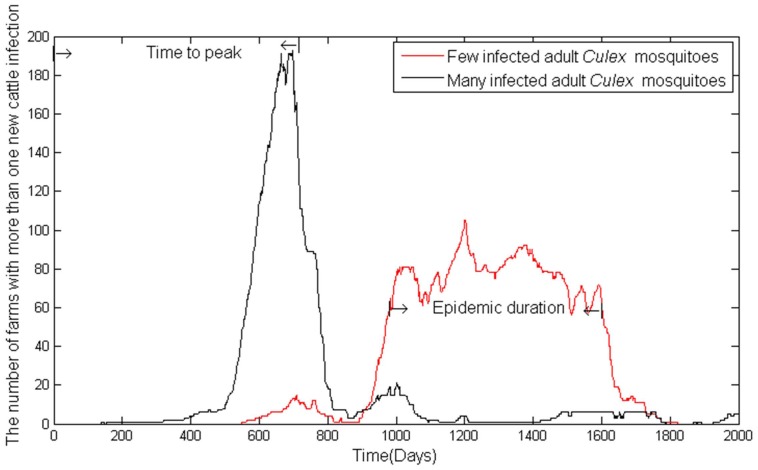
Disease epidemic characteristics based on model output with different numbers of initially infected *Culex* mosquitoes on a small farm. Time to peak infection is the time until the maximal number of cases is observed and epidemic duration is the amount of time an epidemic persists.

Numerical values and definitions are in the Supporting Information. We define that if there is at least one cattle infected, then the farm is infected. 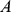 represents the number of infected farms. 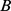 represents the cumulative number of infected cattle throughout simulation. 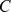 is the total number of infected cattle when the number of infected cattle farms is maximum. 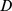 denotes the time to peak number of infected farms, that is, the time it takes from the first day to the day on which the largest number of infected farms appears as shown in Fig. 3. 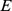 denotes epidemic duration, defined as the number of days with more than 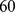 infected cattle farms. The average number of infected farms in each day is in the range of 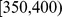, the average cumulative number of infected cattle during simulation is within the range 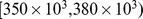, and the average time to peak is within 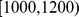.

#### Size of the epidemics

The suffix l or s, (which denote large or small farms) were removed from the initial condition labels when comparing results with different initial infections in the same scale of initial location. The impact of the Rift Valley fever epidemic in terms of infected cattle depends on the size of the initial infection.

When the initial condition of the outbreak is assumed to be *Aedes*-eggs-f (few *Aedes* eggs), the simulations result in a larger cumulative number of infected cattle than the one obtained in the case of *Aedes*-eggs-m (many *Aedes* eggs). When the initial condition of the outbreak is assumed to be *Aedes* -f (few adult *Aedes* mosquitoes), the simulations result in a larger cumulative number of infected cattle than the ones obtained in the case of *Aedes*-m (many adult *Aedes* mosquitoes). Similarly, fewer initial infected *Culex* mosquitoes (*Culex*-f) leads to larger cumulative number of infected cattle than the one obtained in the case of *Culex*-m throughout the simulation period.

When the initial condition of the outbreak is assumed to be Cattle-f (few cattle), the simulations result in a larger cumulative number of infected cattle than the ones obtained in the case of Cattle-m (many cattle).

The total number of infected humans and the total number of farms with at least one infected human remain fewer than one regardless of initial infection conditions. This is likely because the human population of each farm is assumed to be fewer than 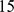. Therefore, human infection is unlikely in this case but this should not be inferred or generalized to be similar in a more heavily populated region or where there are many more persons in direct contact with animals (e.g., slaughter plants).

#### Timing of the epidemics

The temporal characteristics of Rift Valley fever cases followed the general trend that fewer infected individuals in the initial introduction resulted in a delayed epidemic peak. When the initial condition of the outbreak is assumed to be *Aedes*-eggs-f-s, the simulation results in a peak 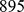 days later than the one with initial starting conditions of *Aedes*-eggs-m-s. When the initial condition of the outbreak is assumed to be *Aedes*-eggs-f-l, the simulations result in a later peak than the *Aedes*-eggs-m-l condition. Comparing another pair of initial conditions, the epidemic peak happens no sooner when few initially infected *Aedes* eggs are considered than when few initial infected *Aedes* adult mosquitoes are assumed. Similarly, the epidemic peak happens not sooner when many initial infected *Aedes* eggs are considered than the one when many initial infected *Aedes* adult mosquitoes are assumed. When the initial condition of the outbreak is assumed to be *Aedes*-f, the simulations result in a later peak than the *Aedes*-l condition. When the initial condition of the outbreak is assumed to be *Culex*-f, the simulations result in a later peak than the *Culex*-l condition. Few initially infected cattle produce a later peak than the one when many cattle are initially infected.

## Discussion

The original meta-population model for Rift Valley fever described by Equations (1) through (20) has been proposed and applied to a case study in two study areas of Texas, the United States. The simulation results are helpful in understanding the mechanisms of RVF virus transmission. Modeling each mosquito species individually requires specific species information to parameterize the model, such as vector competence, which is often not available or is based on assumptions from other species. Therefore, the model groups competent mosquito vectors into two main genera of RVF competent mosquitoes, *Aedes* and *Culex*. The PERT distribution allows for mosquito species of the same genera to be clumped together and for individual variation within a single mosquito species by having a distribution with a most likely value and a range of possible values for each parameter. The distribution also allows the model to be easily adapted to new environments where the vector competence of mosquitoes remains uncharacterized. The model can accommodate various mosquito species of the same genus by adjusting the most likely values and the range of values to account for the variation in vector competence between species. Moreover, the model is not limited to the known mosquito vector species, and newly discovered competent vectors of RVF can be readily included in the model.

The model can be used to study not only local transmission between hosts and vectors, but also trans-location transmission of RVF virus with the network approach. The roles of mosquitoes and livestock in RVF virus transmission can be studied independently because they have separate networks. One infected farm node can spread the infection to other nodes connected to it; therefore, more nodes can be infected over time. The temporal and spatial evolution of RVF virus and its driving force can be analyzed. The spread of RVF virus is estimated within farms as well as between farms, markets, and feedlots. The goal of the simulation analysis is to provide insights into possible pathways for rapid spread of RVF virus among farms and counties. Using the cattle networks, the impact of cattle movement from trade can be investigated as newborn calves mature to weaning and on to harvest. The cattle farms are the source nodes where the cattle are born and raised for several months before being sold through markets or direct to feedlots, or to other farms as stockers or replacement females. Cattle on an infected farm may become infected and then carry the virus to the livestock market or else transition nodes before being sold to another farm, which may introduce the virus to a new farm. On the other hand, infected cattle movement to feedlots (sink nodes) does not propagate the transmission because there is no further transfer of cattle from the nodes except onto slaughter. Different mitigation strategies can be applied according to each node type (source, sink, and transition) within livestock movement network.

Discrete time modeling is appealing in the way it describes the epidemic process, which is conceptualized as evolving through a set of discrete time epochs instead of continuously [39]. Typically infections or illnesses are reported at discrete time (daily or weekly) [39], [40]. Discrete time modeling makes it easier to compare the incidence data with the output of simulations [40]. Moreover, the numerical exploration of discrete time models is more straightforward [40]. Thus, it can be easily implemented [40] by non-mathematicians [40], [41], an advantage in the public health world [40]. Our model allows for simulations of RVF outbreaks on small networks with a few nodes and large scale networks with thousands of nodes. The model is developed not only for the purpose of being applied to the study area of Texas, but also to any geographic region or habitat type of concerns without changing the model. To apply the model to a new study area, the modelers only need to adapt corresponding data into the model. It is time consuming and easy to make mistakes by frequently changing the model to adapt it to a new environment.

In large populations, with a large scale of epidemic incidence, deterministic models can provide good approximations [28]. Moreover, deterministic models are easier to analyze and interpret. However, the given starting condition and fixed parameters of a deterministic model will always result in the same solutions [18] because deterministic models do not reflect the role of chances in disease spread [18]. In principle, stochastic models are more realistic than deterministic models in representing real world activities [28]. In a stochastic model, there are probabilities at each time step transferring from one epidemiological state to another [18]. Hence, the outcomes of different runs may be different [18] and a probability or credibility interval, similar to the confidence interval achieved from statistical analysis of empirical data, can be established. Stochastic models produce quantities such as the probability for an epidemic outbreak to occur and the mean epidemic duration time instead of deterministic results [18]. To reflect the chance of infection more appropriately, a stochastic model will later be developed. However, epidemic outcomes can still be compared with the presented deterministic model applied to case study in the study area of Texas, the United States.

Concerning the discussion of simulation results, *Aedes* are the bridge between *Culex* and livestock starting with *Aedes* egg infection. Infected *Aedes* eggs may hatch infected *Aedes* mosquitoes. The susceptible livestock become infected after being fed on by the infected *Aedes* mosquitoes. *Culex* mosquitoes are amplifiers of RVF virus transmission. *Culex* mosquitoes acquire the infection after blood meals on infected livestock. In return, the infected *Culex* feed on livestock and RVF virus infection is thus amplified. If there are more infected adult mosquitoes at the beginning, whether *Aedes* or *Culex* mosquitoes, the rate of infection is faster, herd immunity is reached faster, the cumulative number of infected cattle is smaller because most recover before they further diffuse to other farms to spread RVF virus, as shown in Fig. 3. If most livestock infected by mosquitoes in a node recover before they move to other nodes, the number of infected livestock and mosquitoes that transmit RVF virus to other nodes is reduced. The eggs do not hatch until their habitats, such as dambos (in Africa) or playas/ponds/sloughs (Texas) are created by rainfall. Moreover, it takes time for *Aedes* eggs to become adult *Aedes* mosquitoes. Consequently, it may take longer to reach the epidemic peak with initially infected *Aedes* eggs than with initially infected *Aedes* mosquitoes.

Cattle can be spreaders of virus because they are frequently bought and sold [42]. Infected cattle may infect a large number of mosquitoes via mosquito bites in a new location. In turn, the infected mosquitoes can bite a large number of susceptible cattle and transmit the virus to them. Movement bans during a RVF outbreak can restrict the further spatial spread of RVF. Therefore, very few infected cattle can infect a large number of susceptible cattle, by interacting with mosquito vectors. Early detection of infected cattle is essential. After local and regional authorities are warned and response planning initiated, such as cattle movement restrictions, culling, insecticide treatments, quarantines, and other methods to limit transmission can also be effective. These methods will be explored in future models. The cumulative number of infected cattle with few infected cattle at the beginning is larger than that with a large number of infected cattle at the beginning. The consequence caused by few initially infected cattle should also be taken seriously.

There are no human cases (integers) in the simulations regardless of initial starting conditions because of the small constant human population in each node of the study region. In high population areas, there can be a large number of human cases. Humans are often exposed to fewer mosquitoes than cattle, especially in more developed countries, which results in lower probability of being infected by mosquitoes. The probability that humans are infected by cattle is also low in this region because the model does not account for contact with the virus via animal slaughter. Hence, the number of infected humans in each farm produced by simulations is fewer than 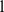. Therefore, an introduction of RVF in the study area of Texas, the United States is likely to be mainly a concern for livestock farms and not an outbreak in humans as recently seen in South Africa based on the deterministic mathematical model presented by [26]. During previous outbreaks, many reported human cases proceeded with livestock cases. In the United States, humans still have the potential of being infected by mosquitoes and livestock especially when many livestock cases are reported. For this reason, the dynamics of human infection during an outbreak and the factors that affect RVF virus transmission will also be studied in future models.

In conclusion, the general epidemiological trend of a smaller initial infection observed through various simulations with various initial staring locations is: 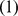 a larger total number of infected cattle, 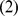 a longer delay after introduction until the peak of the epidemic, and 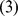 a more prolonged epidemic. If the infection remains small (and possibly undetected) for a longer duration, it expands geographically before the epidemic explodes involving many cattle almost simultaneously. Therefore, an established and endemic condition can generate larger epidemic disease incidence after a long period of apparent hibernation.

## Supporting Information

Table S1 **Quantitative simulation results of different scenarios.**
(PDF)

## References

[1] Daubney R, Hudson JR, Garnham PC (1931) Enzootic hepatitis or Rift Valley fever. an undescribed virus disease of sheep, cattle and man from East Africa. Journal of Pathology and Bacteriology 34: 545–579.

[2] Favier C, Chalvet-Monfray K, Sabatier P, Lancelot R, Fontenille D, et al (2006) Rift Valley fever in West Africa: the role of space in endemicity. Tropical Medicine & International Health 11: 1878–1888. 17176353 10.1111/j.1365-3156.2006.01746.x

[3] Martin V, Chevalier V, Ceccato P, Anyamba A, Simone LD, et al (2008) The impact of climate change on the epidemiology and control of Rift Valley fever. Revue scientifique et technique (International Office of Epizootics) 27: 413–426. 18819669

[4] Chevalier V, Pépin M, Plée L, Lancelot R (2010) Rift Valley fever–a threat for Europe? Eurosurveillance 15: 19506–19517. 20403309

[5] Linthicum KJ, Davies FG, Kairo A, Bailey CL (1985) Rift Valley fever virus (family Bunyaviridae, genus Phlebovirus). isolations from Diptera collected during an inter-epizootic period in Kenya. The Journal of Hygiene 95: 197–209. 2862206 10.1017/s0022172400062434 PMC2129511

[6] Linthicum K, Anyamba A, Tucker C, Kelley P, Myers M, et al (1999) Climate and satellite indicators to forecast Rift Valley fever epidemics in Kenya. Science 285: 397–400. 10411500 10.1126/science.285.5426.397

[7] Zeller H, Fontenille D, Traore-Lamizana M, Thiongane Y, Digoutte J (1997) Enzootic activity of Rift Valley fever virus in Senega. American Journal of Tropical Medicine and Hygiene 56: 265–272. 9129528 10.4269/ajtmh.1997.56.265

[8] Bajardi P, Barrat A, Natale F, Savini L, Colizza V (2011) Dynamical patterns of cattle trade movements. PloS one 6: e19869. 21625633 10.1371/journal.pone.0019869 PMC3097215

[9] Sellers RF, Pedgley DE, Tucker MR (1982) Rift Valley fever, Egypt 1977: disease spread by windborne insect vectors? The Veterinary Record 110: 73–77. 7064329 10.1136/vr.110.4.73

[10] Abdo-Salem S, Waret-Szkuta A, Roger F, Olive MM, Saeed K, et al (2011) Risk assessment of the introduction of Rift Valley fever from the horn of Africa to Yemen via legal trade of small ruminants. Tropical Animal Health and Production 43: 471–480. 20967567 10.1007/s11250-010-9719-7

[11] Davies FG (2006) Risk of a Rift Valley fever epidemic at the haj in Mecca, Saudi Arabia. Revue scientifique et technique (International Office of Epizootics) 25: 137–147. 16796043 10.20506/rst.25.1.1648

[12] European Food Safety Authority (EFSA) (2005) Opinion of the scientific panel on animal health and welfare (AHAW) on a request from the commission related to the risk of a Rift Valley fever incursion and its persistence within the community.

[13] National Institute for Communicable Diseases (2012) Interim report on the Rift Valley fever (RVF) outbreak in South Africa. Available: http://www.nicd.ac.za/?page=rift_valley_fever_outbreak&id=94. Accessed May 23, 2012.

[14] Jouan A, Guenno BL, Digoutte JP, Philippe B, Riou O, et al (1988) An RVF epidemic in southern Mauritania. Ann Inst Pasteur Virol 139: 307–308. 10.1016/s0769-2617(88)80046-7 3207509

[15] World Health Organization. Rift Valley fever in Sudan–Update 4. Available: http://www.who.int/csr/don/2007_11_05/. Accessed April 19, 2012.

[16] Sissoko D, Giry C, Gabrie P, Tarantola A, Pettinelli F, et al (2009) Rift Valley fever, Mayotte, 2007–2008. Emerging Infectious Diseases 15: 568–570. 19331733 10.3201/eid1504.081045 PMC2671425

[17] Kilpatrick AM (2011) Globalization, land use, and the invasion of West Nile virus. Science 334: 323–327. 22021850 10.1126/science.1201010 PMC3346291

[18] Ma S, Xia Y (2009) Mathematical understanding of infectious disease dynamics. World Scientific.

[19] Métras R, Collins LM, White RG, Alonso S, Chevalier V, et al (2011) Rift Valley fever epidemiology, surveillance, and control: what have models contributed? Vector Borne and Zoonotic Diseases 11: 761–771. 21548763 10.1089/vbz.2010.0200

[20] Ross R (1916) An application of the theory of probabilities to the study of a priori pathometry. part I. Proceedings of the Royal Society of London Series A 92: 204–230.

[21] Luz PM, Struchiner CJ, Galvani AP (2010) Modeling transmission dynamics and control of vectorborne neglected tropical diseases. PLoS neglected tropical diseases 4: e761. 21049062 10.1371/journal.pntd.0000761 PMC2964290

[22] Gaff H, Hartley D, Leahy N (2007) An epidemiological model of Rift Valley fever. Electronic Journal of Differential Equations 2007: 1–12.

[23] Mpeshe SC, Haario H, Tchuenche JM (2011) A mathematical model of Rift Valley fever with human host. Acta Biotheoretica 59: 231–250. 21611886 10.1007/s10441-011-9132-2

[24] Niu T, Gaff HD, Papelis YE, Hartley DM (2012) An epidemiological model of Rift Valley fever with spatial dynamics. Computational and mathematical methods in medicine 2012. 10.1155/2012/138757 PMC3424773 22924058

[25] Gao D, Cosner C, Cantrell RS, Beier JC, Ruan S (2013) Modeling the spatial spread of Rift Valley fever in Egypt. Bulletin of mathematical biology 75: 523–542. 23377629 10.1007/s11538-013-9818-5 PMC3664403

[26] Xue L, Scott H, Cohnstaedt L, Scoglio C (2012) A network-based meta-population approach to model Rift Valley fever epidemics. Journal of Theoretical Biology 306: 129–144. 22564391 10.1016/j.jtbi.2012.04.029

[27] Manore C, McMahon B, Fair J, Hyman JM, Brown M, et al (2011) Disease properties, geography, and mitigation strategies in a simulation spread of rinderpest across the United States. Veterinary research 42: 55. 21435236 10.1186/1297-9716-42-55 PMC3072946

[28] Keeling M, Rohani P (2008) Modeling infectious diseases in humans and animals. Princeton University Press.

[29] Dominguez BJ (2007) Characterization of livestock herds in extensive agricultural settings in southwest Texas. Master’s thesis, Texas A&M University, U.S.A.

[30] United States Department of Agriculture (2007) 2007 census publications. Available: http://www.agcensus.usda.gov/Publications/2007/Full_Report/Census_by_State/Texas/index.asp. Accessed April 15, 2012.

[31] Riley S (2010) Coping without farm location data during a foot-and-mouth outbreak. Proceedings of the National Academy of Sciences of the United States of America 107: 957–958. 20080603 10.1073/pnas.0913286107 PMC2824299

[32] Otero M, Solari H (2010) Stochastic eco-epidemiological model of dengue disease transmission by Aedes aegypti mosquito. Mathematical Biosciences 223: 32–46. 19861133 10.1016/j.mbs.2009.10.005

[33] Turell MJ, Wilson WC, Bennett KE (2010) Potential for North American mosquitoes (Diptera: Culicidae) to transmit Rift Valley fever virus. Journal of medical entomology 47: 884–889. 20939385 10.1603/me10007

[34] Wonham MJ, Lewis MA, Renclawowicz J, van den Driessche P (2006) Transmission assumptions generate conicting predictions in host-vector disease models: a case study in West Nile virus. Ecology Letters 9: 706–725. 16706915 10.1111/j.1461-0248.2006.00912.x

[35] Gong H, Degaetano AT, Harrington LC (2010) Climate-based models for West Nile Culex mosquito vectors in the Northeastern US. International Journal of Biometeorology 55: 435–446. 20821026 10.1007/s00484-010-0354-9

[36] Rueda LM, Patel KJ, Axtell RC, Stinner RE (1990) Temperature-dependent development and survival rates of Culex-quinquefasciatus and Aedes-aegypti (Diptera: Culicidae). Journal of medical entomology 27: 892–898. 2231624 10.1093/jmedent/27.5.892

[37] National Climatic Center. NOAA Satellite and Information Service. Accessed April 18, 2012.

[38] Linacre E (1977) A simple formula for estimating evaporation rates in various climates, using temperature data alone. Agricultural Meteorology 18: 409–424.

[39] Longini IM (1986) The generalized discrete-time epidemic model with immunity: a synthesis. Mathematical biosciences 82: 19–41.

[40] Brauer F, Feng Z, Castillo-Chavez C (2010) Discrete epidemic models. Mathematical biosciences and engineering 7: 1–15. 20104945 10.3934/mbe.2010.7.1

[41] Katriel G (2013) Stochastic discrete-time age-of-infection epidemic models. International Journal of Biomathematics 6: 125066.

[42] Arino J, Jordan R, van den Driessche P (2007) Quarantine in a multi-species epidemic model with spatial dynamics. Mathematical biosciences 206: 46–60. 16343557 10.1016/j.mbs.2005.09.002

[43] Canyon DV, Hii JLK, Muller R (1999) The frequency of host biting and its e_ect on oviposition and survival in Aedes aegypti (Diptera: Culicidae). Bulletin of entomological research 89: 35–39.

[44] Hayes RO, Tempelis CH, Hess AD, Reeves WC (1973) Mosquito host preference studies in Hale county, Texas. American Journal of Tropical Medicine and Hygiene 22: 270–277. 4143926 10.4269/ajtmh.1973.22.270

[45] Jones CJ, Lloyd JE (1985) Mosquitos feeding on sheep in southeastern Wyoming. Journal of the American Mosquito Control Association 1: 530–532. 2906690

[46] Magnarelli LA (1977) Host feeding patterns of Connecticut mosquitos (Diptera-Culicidae). American Journal of Tropical Medicine and Hygiene 26: 547–552. 17310 10.4269/ajtmh.1977.26.547

[47] Pratt HD, Moore CG (1993) Vector-borne disease control: mosquitoes, of public health importance and their control. U.S. Department of Health and Human Services, Atlanta, GA.

[48] Turell MJ, Kay BH (1998) Susceptibility of selected strains of Australian mosquitoes (Diptera: Culicidae) to Rift Valley fever virus. Journal of medical entomology 35: 132–135. 9538572 10.1093/jmedent/35.2.132

[49] Turell MJ, Faran ME, Cornet M, Bailey CL (1988) Vector competence of senegalese Aedes fowleri (Diptera: Culicidae) for Rift Valley fever virus. Journal of Medical Entomology 25: 262–266. 3404545 10.1093/jmedent/25.4.262

[50] Turell MJ, Bailey CL (1987) Transmission studies in mosquitoes (Diptera: Culicidae) with disseminated Rift Valley fever virus infections. Journal of Medical Entomology 24: 11–18. 2880998 10.1093/jmedent/24.1.11

[51] Wekesa JW, Yuval B, Washino RK (1997) Multiple blood feeding by Anopheles freeborni and Culex tarsalis (Diptera: Culicidae): Spatial and temporal variation. Journal of Medical Entomology 34: 219–225. 9103766 10.1093/jmedent/34.2.219

[52] Erasmus BJ, Coetzer JAW (1981) The symptomatology and pathology of Rift Valley fever in domestic animals. Contrib Epidemiol Biostat 3: 77–82.

[53] Bates M (1949) The natural history of mosquitoes. MacMillan, New York. 378p.

[54] Moore CG, McLean RG, Mitchell CJ, Nasci RS, Tsai TF, et al.. (1993) Guidelines for arbovirus surveillance programs in the United States. Center for Disease Control and Prevention.

[55] Radostits OM (2001) Herd healthy: food animal production medicine. Saunders, third edition.

[56] Peters CJ, Linthicum KJ (1994) Rift Valley fever. In: Handbook of Zoonoses. Second Edition. Section B: Viral. G.B. Beran (Ed.), Boca Raton, Fl: CRC Press, Inc. 125–138.

[57] Freier JE, Rosen L (1987) Vertical transmission of dengue viruses by mosquitoes of the Aedes scutellaris group. The American Journal of Tropical Medicine and Hygiene 37: 640–647. 3688318 10.4269/ajtmh.1987.37.640

