# Supplementary material for: A Hierarchical Network Approach for Modeling Rift Valley Fever Epidemics with Applications in North America
Source: PLoS One. 2013 May 7;8(5):e62049. doi: 10.1371/journal.pone.0062049 (PMC3646918; doi:10.1371/journal.pone.0062049)
Supplement: Table S1 — Quantitative simulation results of different scenarios. (PDF) [file pone.0062049.s001.pdf]

Supporting Information

The quantitative simulation results of different scenarios is shown in Table $S1$.

Table S1. Quantitative simulation results of different scenarios. We define that if there is at least one cattle infected, then the farm is infected. The number of infected farms is represented by A and the cumulative number of infected cattle throughout simulation is represented by B. The total number of infected cattle when the number of infected cattle farms is maximum (C). The time to peak number of infected farms (D) means the time it takes from the first day to the days on which the largest number of infected farms appears. Epidemic duration (E) means the period with more than 60 infected cattle farms. The peak number of farms with more than one infected human is represented by F and the peak number of infected humans in a single farm in a day is represented by G. The total number of farms is 3526 and the total number of cattle in all farms is 303240.

Farm size	Size of initial infection	Outcome characteristics	Initial source of infection			
			Aedes eggs	Adult *Aedes*	Adult *Culex*	Cattle
Small	few	A	359	319	267	183
		B	410×10^3	411×10^3	397×10^3	374×10^3
		C	16288	6369	4230	2557
		D	1596	1382	1205	1012
		E	444	471	592	291
		F	0	0	0	0
		G	0	0	0	0
	many	A	224	437	610	388
		B	364×10^3	335×10^3	313×10^3	343×10^3
		C	1772	3125	4433	2773
		D	701	701	700	701
		E	278	181	217	227
		F	0	0	0	0
		G	0	0	0	0
Large	few	A	293	197	296	342
		B	407×10^3	382×10^3	354×10^3	413×10^3
		C	2907	3878	2459	4411
		D	1205	1204	711	1382
		E	557	443	278	467
		F	0	0	0	0
		G	0	0	0	0
	many	A	631	732	745	208
		B	315×10^3	321×10^3	332×10^3	385×10^3
		C	4251	4689	4428	3778
		D	700	655	608	1204
		E	226	260	276	449
		F	0	0	0	0
		G	0	0	0	0

The outcome characteristics are classified into the following ranges.

very small ($0 < A < 300$ or $0 < B < 320 \times 10^3$ or $0 < C < 3000$),
small ($300 \leqslant A < 350$ or $320 \times 10^3 \leqslant B < 350 \times 10^3$ or $3000 \leqslant C < 4000$),
average ($350 \leqslant A < 400$ or $350 \times 10^3 \leqslant B < 380 \times 10^3$ or $4000 \leqslant C < 4500$),
large ($400 \leqslant A < 600$ or $380 \times 10^3 \leqslant B < 400 \times 10^3$ or $4500 \leqslant C < 6000$),
very large ($A \geqslant 600$ or $B \geqslant 400 \times 10^3$ or $C \geqslant 6000$),
very short or really large ($0 < D < 700$ or $0 < E < 250$),
short ($700 \leqslant D < 1000$ or $250 \leqslant E < 300$),
medium ($1000 \leqslant D < 1200$ or $300 \leqslant E < 450$),
long ($1200 \leqslant D < 1300$ or $450 \leqslant E < 500$),
very long ($D \geqslant 1300$ or $E \geqslant 500$).
